# Chryseobacterium indologenes, an Emerging Bacteria: A Case Report and Review of Literature

**DOI:** 10.7759/cureus.6720

**Published:** 2020-01-21

**Authors:** Dora E Izaguirre-Anariba, Vel Sivapalan

**Affiliations:** 1 Infectious Disease, Columbia University at Harlem Hospital Center, New York, USA

**Keywords:** c. indologenes, bacteremia, central line-associated infections (clabsi), multi-drug resistant bacteria

## Abstract

Chryseobacterium indologenes are aerobic, Gram negative, nonfermentative rods that are intrinsically multi-drug resistant. Reported infections include bacteremia, pneumonia, meningitis, myositis, keratitis, and indwelling devices. We present the clinical course of a 52-year-old African male with a medical history of end stage renal disease (ESRD) in hemodialysis with multiple episodes of central line-associated bloodstream infections (CLABSI) presenting with symptoms of chills, malaise, and localized erythema on insertion site of permacath. Blood cultures obtained from catheter showed C. indologenes. Successful response was obtained with piperacillin/tazobactam based on sensitivity and removal of indwelling catheter. Given the increase in the number of cases reported in the literature, guidelines for the management of this pathogen should be considered.

## Introduction

Chryseobacterium species are Gram negative, aerobic, nonfermentative, oxidase-positive, catalase-positive, and non-motile bacilli [1-2]. They produce a distinctive yellow to orange pigment on blood agar [3-4].

The most frequent isolates of Chryseobacterium species include *Chryseobacterium indologenes*, *C. meningosepticum*, and *C. gleum*. *C. indologenes* was formerly known as flavobacterium indologenes. Bacteremia due to *C. indologenes* was first described in Taiwan by Hsue et al. [5]. It is found naturally in soil, water, plants, and food products. It is not normally found in the human microflora [4, 6]. In the hospital setting, *C. indologenes* may be found in water systems and on wet surfaces which serve as a potential reservoir of infection. It resists chlorination and can survive in municipal water supplies [7].

Nosocomial infections due to *C. indologenes* have been linked to the use of indwelling devices during a hospital stay. Additionally, the colonization of patients through contaminated medical devices involving fluids such as respirators, endotracheal tubes, mist tents, humidifiers, incubator for newborns, ice chests, and syringes has been reported [3, 7-8].

Most common related infections include bacteremia, pneumonia, meningitis, pyomyositis, keratitis, and also indwelling device-associated infections such as urinary tract, surgical, and burn wound infections [2].

The clinical significance of *C. indologenes* has not yet been established as it has not been frequently recovered from clinical specimens. Most strains of *C. indologenes* do not grow on MacConkey agar, but grow well on blood agar after 24 h incubation at 37°C [3-4]. 

This bacteria produces a biofilm on foreign materials (i.e., indwelling devices) and protease activity that may play an important role in the virulence of invasive infections [4, 9]. *C. indologenes* was first reported in 1993 by Bhagawati et al. in a patient presenting with ventilator-associated pneumonia [10]. Later, more cases presenting as bacteremia, pneumonia, meningitis, pyomyositis, keratitis, and also contaminated surgically implanted devices have been reported [1, 6, 8, 11]. Appropriate choice of antimicrobial agents is challenging due to the unpredictability and multiple drug resistance of this microorganism to antibiotics [3]. 

## Case presentation

**Figure 1:**
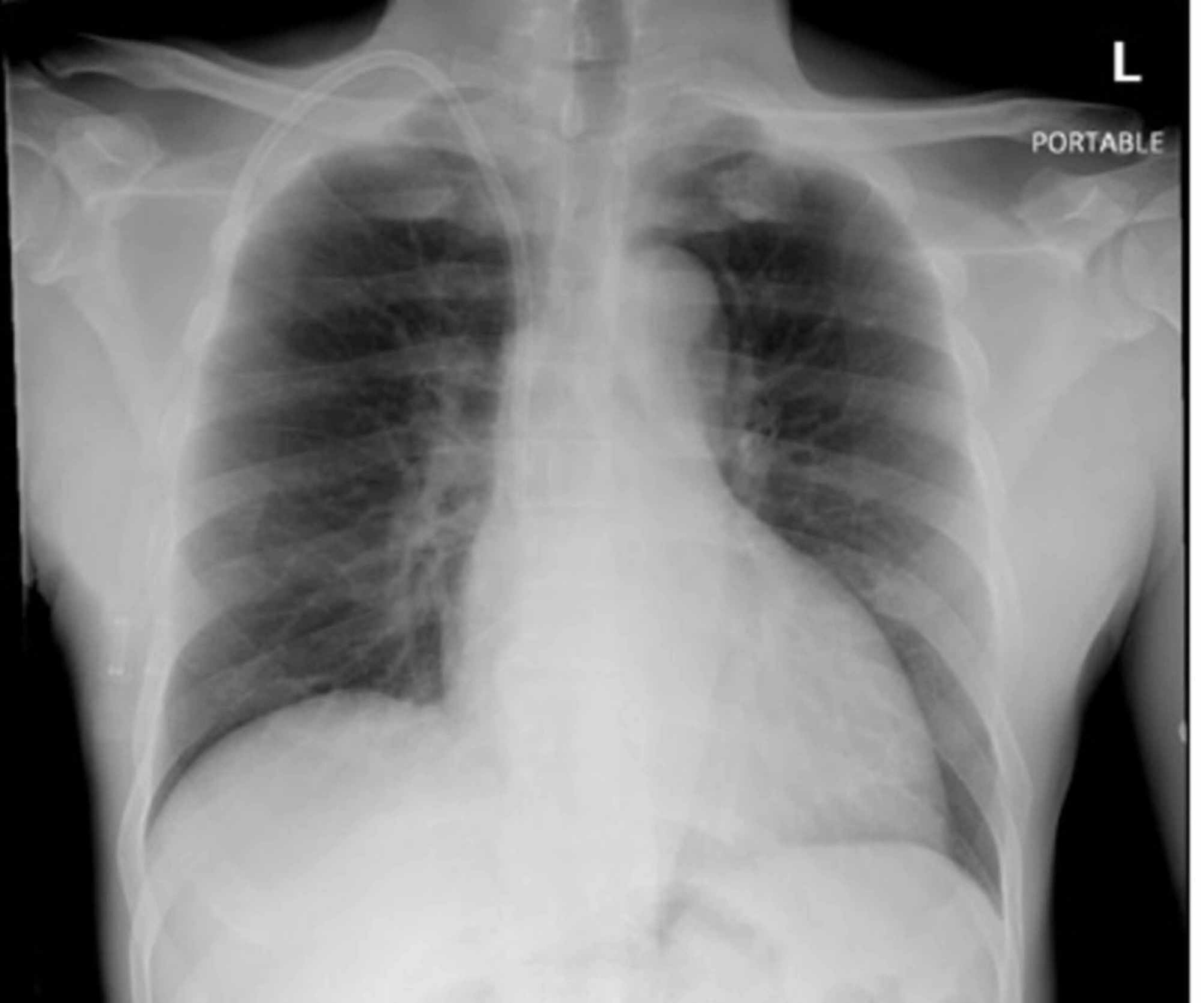
Chest X-ray. Right internal jugular dialysis catheter with the tip projecting to the right cavoatrial junction. No evidence of acute cardiopulmonary disease.

**Figure 2:**
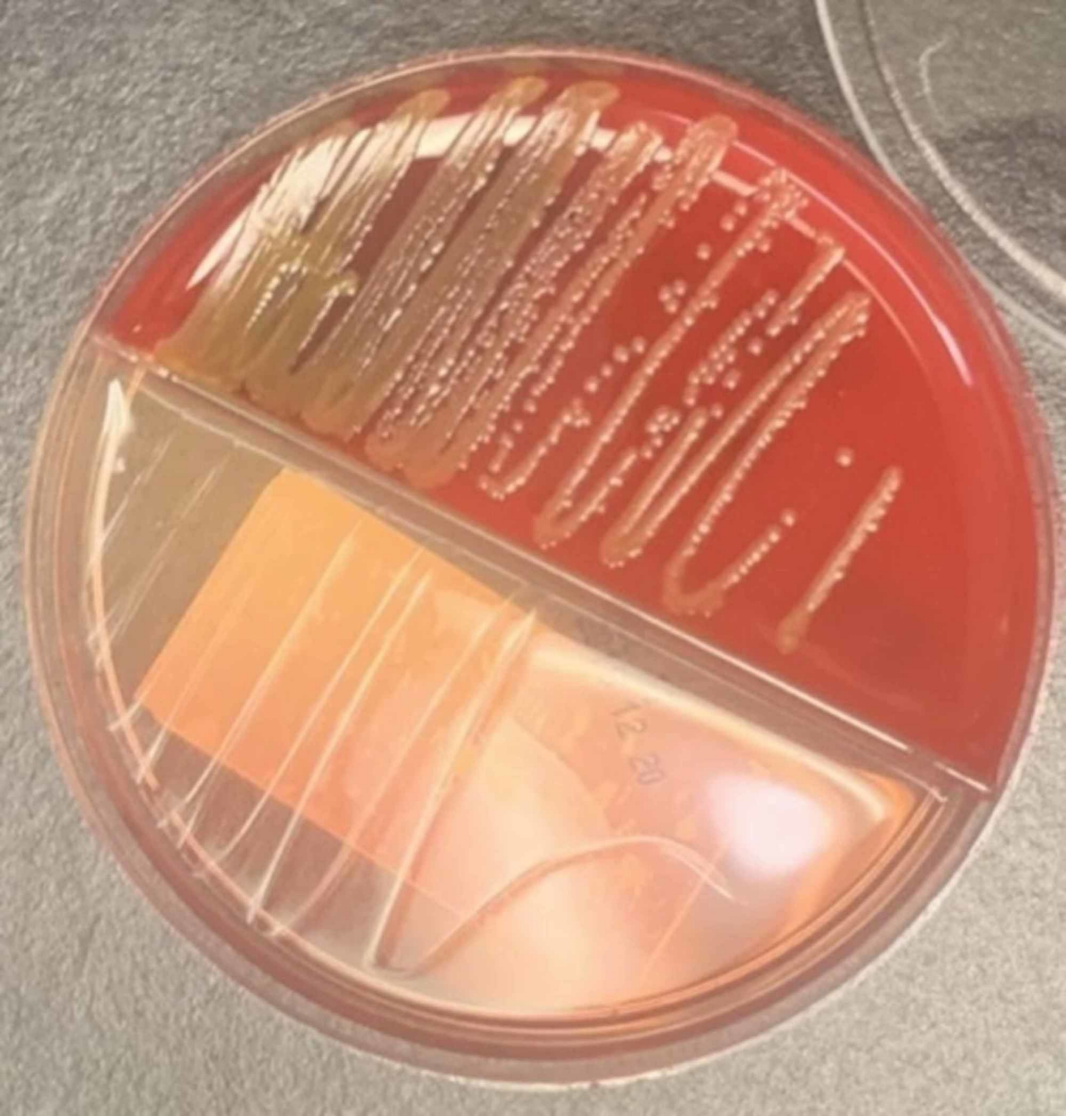
Subculture of isolated C. indologenes in blood agar (red) and MacConkey agar (clear). *C. indologenes* grows yellowish orange colonies in blood agar. Note that there is no growth of the bacteria in MacConkey agar.

A 52-year-old man from Senegal (West Africa) with past medical history of hypertension, depression, end stage renal disease (ESRD) in hemodialysis for 10 months and two previous admission due to sepsis due to central line-associated bloodstream infection (CLABSI) with Acinetobacter baumannii was treated lastly with cefepime based on culture and sensitivity reports. He had his permacath catheter removed the month prior to his admission. Subsequent blood cultures were obtained negative; however, months later he was found with blood cultures growing bacteria while still receiving cefepime for previous CLABSI. Hence, he was referred by his nephrologist for evaluation and admission. At that time, the patient was treated with meropenem based on preliminary reports until obtaining final reports of new cultures. The patient complained only of chills, general malaise, and local itchiness on the insertion site of newly placed permacath. No sepsis was evidenced on arrival. His physical examination was unremarkable. The insertion site of his permacath was noted dry and clean with mild erythema. Initial blood work revealed white cell count of 6190/mcl, hemoglobin 11.1 g/dL, neutrophils 63%, lymphocytes 15%, eosinophils 6%, BUN 39 mg/dL, and creatinine 9.1 mg/dL. His chest radiograph did not demonstrate evidence of ongoing acute infectious process (Figure 1). The patient received meropenem 500 mg daily (for two days), dose adjusted in patients receiving intermittent hemodialysis. His hemodialysis catheter was removed. New cultures from peripheral sites (two sets taken on arrival and one taken by hemodialysis center) grew *C. indologenes* (Figure 2) resistant to carbapenems and only sensitive to quinolones (ciprofloxacin and levofloxacin), trimetropin sulfametoxazol, and piperacillin-tazobactam (Table 1). Consequently, it was recommended to start the patient on levofloxacin which was contraindicated due to abnormal electrocardiogram changes (Q-T segment prolongation); therefore, piperacillin-tazobactam 2.25 g every 8 h was initiated to complete 10 days of therapy. During his hospital stay the patient received his hemodialysis with a temporary shiley catheter, and by the time of his discharge he had a new permacath in place. Two more sets of blood cultures repeated prior discharge were negative. Transthoracic echocardiogram performed was reassuring with no evidence of vegetations.

**Table 1:** Blood culture report. Component final growth in aerobic bottle: *C. indologenes* (carbapenem resistant) Gram stain growth in aerobic bottle: Gram negative rods Organism: *C. indologenes* multi-drug resistant organisms (MDRO) (POSITIVE)

Susceptibility	Chryseobacterium indologenes (Carbapenem resistant)
Amikacin	>32 Resistant
Aztreonam	>16 Resistant
Cefepime	>16 Resistant
Ceftriaxone	>32 Resistant
Ciprofloxacin	<=1 Sensitive
Gentamicin	>8 Resistant
Levofloxacin	<=2 Sensitive
Meropenem	>8 Resistant
Piperacillin/Tazobactam	16 Sensitive
Tobramycin	>8 Resistant
Trimethoprim/Sulfa	<=2/38 Sensitive

After discharge, the patient has not had recurrent episodes of CLABSI. He was educated about maintaining proper care of his hemodialysis catheter.

## Discussion

*Chryseobacterium indologenes* is a nonmotile, Gram negative bacilli that is intrinsically resistant to aminoglycosides, first-generation cephalosporins, aminopenicillins, and aztreonam [12]. The increase in clinical usage of colistin and tigecycline against emerging carbapenem-resistant pathogens has been associated with significant problems in the critical care setting [3, 9].

Alon et al. (2018) presented a cohort of seven patients who presented bacteremia with *C. indologenes* in which most common characteristics of these patients were immunocompromised state with multiple comorbidities that have undergone a surgical procedure. They also reported similar resistance sensitivity patterns to previous reports in the literature. The most potent agents reported against *C. indologenes* are quinolones (gatifloxacin and levofloxacin) and trimethoprim sulfamethoxazole (>95% susceptibility). Ciprofloxacin, cefepime, ceftazidime, piperacillin, and rifampin showed significant susceptibility. Furthermore, as per the SENTRY antimicrobial surveillance program (1997-2001), vancomycin, chloramphenicol, linezolid, and glycopeptides are not appropriate choices for treating infections due to this organism [8, 10]. In most cases, duration of therapy for bacteremia ranges from 7 to 14 days. Our patient responded successfully to the regimen received; therefore, the decision was made to provide a 10-day course of antibiotics. Although our patient has his permacath catheter removed some researchers have reported eradication of bacteria without removal of indwelling catheters [9]. Arteriovenous fistula (AVF) creation was initially attempted but due to multiple episodes of CLABSI, this procedure had been deferred until stable resolution of his bacteremia. Suggested mortality rate associated to infections with *C. indologenes* is around 17% [6].

## Conclusions

Although *C. indologenes* is an uncommon pathogen, the number of cases reported has increased throughout the years. This increase is probably the result of the improvement of diagnostic modalities. However, there are no guidelines for the treatment of patients presenting with these infections. In patients with no bloodstream infections, the removal of indwelling devices may not be clinically indicated with some successful cases reported in the literature.
